# Neuroinflammation in long-term cognitive impairment after aneurysmal subarachnoid hemorrhage

**DOI:** 10.1177/17474930251362004

**Published:** 2025-07-15

**Authors:** Reinier WP Tack, Nelleke Tolboom, Bas Meyer Viol, Sandeep SV Golla, Bart NM van Berckel, Irene C van der Schaaf, Ronald Boellaard, Alberto de Luca, Martine JE van Zandvoort, Johanna MA Visser-Meily, Elly M Hol, Gabriel JE Rinkel, Mervyn DI Vergouwen

**Affiliations:** 1 Department of Neurology and Neurosurgery, UMC Utrecht Brain Center, University Medical Centre Utrecht, Utrecht University, Utrecht, The Netherlands; 2 Department of Radiology and Nuclear Medicine, University Medical Center Utrecht, Utrecht University, Utrecht, The Netherlands; 3 Department of Radiology and Nuclear Medicine, Amsterdam UMC, VU University Medical Center, Amsterdam, The Netherlands; 4 Image Sciences Institute, Division of Imaging and Oncology, University Medical Center Utrecht, Utrecht University, Utrecht, the Netherlands; 5 Department of Rehabilitation, and Center of Excellence for Rehabilitation Medicine, UMC Utrecht Brain Center, University Medical Centre Utrecht, Utrecht University, Utrecht, The Netherlands; 6 Department of Translational Neuroscience, UMC Utrecht Brain Centre, University Medical Centre Utrecht, Utrecht University, Utrecht, The Netherlands

**Keywords:** Subarachnoid hemorrhage, cognitive Impairment, neuroinflammation

## Abstract

**Background:**

Survivors of aneurysmal subarachnoid hemorrhage (aSAH) often have cognitive impairment, which may be caused by long-term inflammation. We aimed to determine whether long-term neuroinflammation or microstructural brain damage is associated with cognitive impairment after aSAH.

**Methods:**

In this prospective cohort study, we included patients >3 years after aSAH between 2020 and 2022. Patients underwent neuropsychological evaluation, translocator protein 18 kDA (TSPO) positron emission tomography (PET) imaging using [^18^F]DPA-714 to determine neuroinflammation, and brain diffusion kurtosis imaging (DKI) to determine microstructural damage. We compared TSPO PET binding potential, mean kurtosis (MK), kurtosis anisotropy (KA), axial kurtosis (AK), and radial kurtosis (RA) between groups and determined which metric was correlated with individual cognitive tests.

**Results:**

We included 27 patients with aSAH; 14 with and 13 without cognitive impairment. Whole-brain TSPO binding potential was similar between groups (mean BP_ND_: −0.046 [95% confidence interval (CI): −0.105; 0.013] vs −0.047 [95% CI −0.108; 0.014], p = 0.98) and there were no regional differences. Those with cognitive impairment had a lower whole-brain MK (mean MK 0.70 [95% CI: 0.69-0.72] vs 0.73 [95% CI: 0.72-0.74], p = 0.03) and whole-brain AK (mean AK 0.81 [95% CI: 0.78-0.83] vs 0.86 [0.84-0.87], p = 0.04). Left thalamic MK and AK were correlated with tests of verbal memory (r = 0.60-0.67, p < 0.01), while other correlation tests were non-significant.

**Conclusion:**

Our results do not support the hypothesis that long-term cognitive impairment after aSAH is caused by long-term neuroinflammation. Instead, microstructural damage may play a role.

## Graphical abstract

**Graphical abstract:**
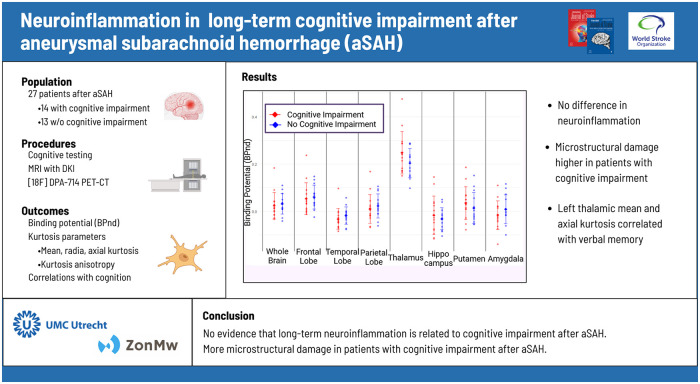

## Introduction

Aneurysmal subarachnoid hemorrhage (aSAH) is a devastating subtype of stroke, with a case-fatality rate of around 40%. Cognitive impairment occurs in approximately three out of four survivors and mainly consists of deficits in memory, executive functioning, and language.^1^ Cognitive impairment after aSAH negatively affects functional status, emotional health, participation, and quality of life.^2^ While several clinical and radiological parameters have been found to be associated with cognitive impairment following aSAH, the pathophysiology remains unknown.^3^ As the prevalence of cognitive impairment is high even in those without radiological abnormalities and with good functional outcomes, it has become imperative to explore the underlying pathophysiology of cognitive impairment after aSAH and with that the potential prognostic or therapeutic implications.^4^

Several studies suggest that neuroinflammation, in particular microglia activation, plays a role in the pathophysiology of cognitive impairment after aSAH.^5,6^ ASAH triggers an acute and potentially prolonged inflammatory response, likely driven by multiple factors. Blood breakdown products (e.g. hemoglobin, heme, iron), thrombin, and other coagulation cascade elements are known to be potent activators of microglia. In addition, early brain injury and delayed cerebral ischemia, may contribute to neuroinflammation. This leads to subsequent astrocyte activation and a cascade involving pro-inflammatory cytokine release.^7^ In rodent models of SAH modeled through hematoma injection or endovascular perforation, this neuroinflammatory response persists for at least 28 days after the bleeding and has been shown to coincide with neuro-axonal injury, potentially explaining its role in the pathophysiology of cognitive impairment.^8^ However, there are no data from studies in humans supporting the hypothesis that long-term neuroinflammation occurs after aSAH and whether it is associated with cognitive impairment.

Novel imaging modalities offer a unique insight into brain pathology. Translocator protein 18 kDA (TSPO) is an in vivo marker of neuroinflammation and can detect microglial and astrocyte activation in several neurological diseases, such as multiple sclerosis and Alzheimer’s disease.^9^ [^18^F]DPA-714 is a second-generation TSPO positron emission tomography (PET) tracer with favorable binding characteristic compared with other TSPO PET ligands.^10^ Brain magnetic resonance imaging (MRI) with diffusion kurtosis imaging (DKI) can be used to detect microstructural damage.^11^ Using [^18^F]DPA-714 PET and DKI, we aimed to determine whether long-term neuroinflammation is increased in aSAH survivors with cognitive impairment compared to those without such impairment, and whether long-term neuroinflammation or microstructural damage is associated with cognitive performance.

## Methods

This prospective cohort study was performed between November 2020 and February 2022. We used our institutional aSAH database with data from a previous genome-wide association study to contact patients with a >3-year history of aSAH.^12^ We included only patients more than 3 years after aSAH to minimize the influence of ongoing recovery or subacute complications, and to focus on stable, long-term cognitive outcomes. As the rs6971 polymorphism in exon 4 of the TSPO gene has been shown to affect the binding potential of TSPO tracers, we contacted only those patients who were high affinity [^18^F]DPA-714 binders based on rs6971 genotyping.^13^

### Inclusion and exclusion criteria

The inclusion criteria were (1) history of aSAH > 3 years prior to inclusion; (2) functional independence (defined as modified Ranking Scale (mRS) of 0–2) at time of inclusion; (3) age 18 years or older at time of aSAH; and (4) high-affinity binders on rs6971 genotyping. Exclusion criteria were (1) inability to undergo MRI or PET scanning (due to a pacemaker, claustrophobia, or severe back pain); (2) pregnancy; (3) history of ischemic stroke or intracerebral hemorrhage; and (4) clinically diagnosed dementia.

### Study schedule

Included patients were scheduled for three visits. During the first visit, patients underwent neuropsychological evaluation to determine cognitive status. Six weeks later, during the second visit, a brain MRI scan was made. During the third visit, after another 2–6 weeks, a TSPO PET scan with [^18^F]DPA-714 was performed. Demographics, medical history, medication use, and presence of mild cognitive impairment (defined as mild cognitive decline expressed by the individual, reliable informant or clinician, mirrored by objective cognitive assessment) was obtained through electronic health records of included patients.^14^ Follow-up at our tertiary referral center includes extensive cognitive assessment in all patients between 6 and 12 weeks following aSAH, which allowed determination of cognitive status prior to study inclusion. Matching between patients with and without cognitive impairment was performed on age and time after aSAH to ensure matched individuals were within a 5-year age range and within 1-year difference in time after aSAH. Inclusion was halted when our sample with available rs6971 genotyping was exhausted.

### Neuropsychological evaluation

The neuropsychological examination was performed by researchers trained by an experienced neuropsychologist (M.J.E.Z.) and consisted of the following 11 subtests: the Digit Span Test of the Wechsler Adult Intelligence Scale (WAIS) IV, the Rey Auditory Verbal Learning Task (RAVLT)-Dutch version immediate and delayed recall, the Rey–Osterrieth Complex Figure Test (CFT) copy and delayed recall, the Symbol Substitution Test (SST) of the WAIS III, the Category Fluency Test, the Letter Fluency Test, the Go/No-Go task of the Frontal Assessment Battery (FAB), the Boston Naming Test (BNT), and the Visual Elevator Task. These subtests covered the following five cognitive domains: language, memory, executive functioning, attention, and visuospatial functioning. Individual subtest scores were corrected for age, sex, and education level using norm-scores and transformed into percentiles. Performance on individual tests was defined as “slightly impaired” at a score ⩽16th percentile and impaired at a score ⩽5th percentile. As our neuropsychological battery was shorter than previously described batteries to increase patient inclusion,^15^ we used a definition of cognitive impairment as having at least one “slightly impaired” and one “impaired” score in at least two out of five cognitive domains. We further computed a global cognitive score for each patient using the average z-score across all cognitive subtests.

### MRI data acquisition and analysis

MRI imaging was performed on a Philips 3.0 Tesla scanner (Interna; Philips, Best, The Netherlands). MRI sequences obtained were 3D T1, T2 fluid-attenuated inversion recovery (FLAIR) and diffusion tensor imaging (DTI) sequences. A trained neuroradiologist visually examined MRI sequences to determine pre-existing ischemic lesions. Diffusion-weighted MRI data were acquired using a single-shot spin echo-planar imaging sequence with the following parameters: Repetition time 7500 ms, Echo time 55 ms, Field of View matrix 112 × 112, flip angle 90°, 66 diffusion encoding gradients, high b-values 1000 and 2000 s/mm^2^, with phase encoding direction anterior to posterior and acquired voxel size 2 mm × 2 mm × 2 mm. The acquisition time was 13 min and 30 s. Data processing was performed using the ExploreDTI (exploredti.com) software package. Initially, DWIs were corrected for signal drift, motion, Eddy current-induced distortions, and Gibbs ringing.^16,17^ Kurtosis parameters were computed using the Robust Extraction of Kurtosis INDices with Linear Estimation (REKINDLE) approach.^18^ Region of interest (ROIs) mean kurtosis (MK), kurtosis anisotropy (KA), axial kurtosis (AK), and radial kurtosis (RK) values were obtained using automated atlas-based analysis with our adjusted Hammers atlas.^19^

### PET acquisition and analysis

PET scans were performed on a Biograph VISION PET-CT scanner (Siemens Healthineers, The Netherlands). Radiosynthesis of [^18^F]DPA-714 was performed in the Amsterdam University Medical Center, location VUMC, as described earlier.^20^ After radioactive transportation to the UMCU, quality controls were performed, and radiotracer was prepared in the radionucleotide lab of our institution. After low-dose CT scan for attenuation purposes, a bolus of 250± 24 MBq [^18^F]DPA-714 was injected intravenously, followed by 60-min dynamic PET acquisition and reconstruction as described earlier.^21^ Images were reconstructed with a matrix size of 400 × 400 and zoom factor of 1, resulting in a voxel size of 1.17 × 1.17 × 3.27 mm³. Quantitative analysis of PET images was performed using PMOD software (version 4.105, Zürich, Switzerland).

For each patient, MRI data were co-registered to the [^18^F]DPA-714 image. Voxel wise time activity curves were regionally averaged by superimposing the MR-derived ROIs from our adjusted Hammers atlas onto the dynamic PET images. Regional and whole-brain binding potential (BP_ND_) was derived using a simplified reference tissue model (SRTM), where cerebellar gray matter was used as a pseudo-reference area as described earlier.^20^

### Determination of regions of interest

In both MRI and PET-imaging, an adjusted version of the Hammers atlas was used for ROI analyses.^19^ This atlas includes separate labels for gray and white matter, allowing for tissue-specific quantification without additional masking. Predetermined regions of interest and corresponding regions within this atlas were: thalamus, hippocampus, amygdala, putamen, frontal lobe gray matter (consisting of middle frontal gyrus, precentral gyrus, gyrus rectus, orbito-frontal gyrus, inferior frontal gyrus, and superior frontal gyrus), temporal lobe gray matter (consisting of anterior medial temporal lobe, anterior lateral temporal lobe, ambient and parahippocampal gyri, superior temporal gyrus, inferior middle temporal gyri, fusiform gyrus, and posterior temporal lobe), and parietal lobe gray matter (consisting of postcentral gyrus, superior parietal gyrus, and inferior lateral parietal lobe). White matter of frontal, temporal and parietal Lobe were calculated separately. Whole-brain and bilateral regional values were calculated using a volume-weighted average of measurements.

### Validation of reference region for quantification of TSPO binding

Previous research has shown that a true reference region devoid of any TSPO binding does not exist.^22^ In healthy controls and Alzheimer’s disease (AD) patients, cerebellar gray has been demonstrated to be suitable as a pseudo-reference region.^23^ To determine whether cerebellar gray matter would be suitable as a reference region in our population, we performed a second PET acquisition in seven consecutive post-aSAH patients with cognitive impairment between 135 and 155 min post-injection. We calculated standardized uptake values based on body surface area (SUVBSA) of cerebellar gray matter and compared the values with those derived from historical controls using a plasma input model in previous research.^20^

### Statistical analysis

Descriptive statistics were performed to describe the demographics, clinical characteristics, and results of neuropsychological assessment. Mean with standard deviation (SD), median with first and third quartile (Q1–Q3), and number and proportion are used where appropriate. Comparison between groups was performed using independent samples T-test, Mann–Whitney U test, and chi-square test, where appropriate. We compared imaging metrics between patients with and without cognitive impairment: first, we calculated mean whole-brain and regional TSPO BP_ND_, and compared these through independent samples t-test. False discovery rate (FDR) correction was applied for multiple comparisons across brain regions. A sensitivity analysis was performed excluding brain regions with infarction on T2 or FLAIR MRI. A second sensitivity analysis was performed, in which we compared means of white matter TSPO BP_ND_ in frontal, temporal and parietal lobes. To ensure robustness, we further performed linear regression of the global cognitive score onto our regional BP_ND_ across all included patients. We compared whole-brain and regional MK, KA, AK, and RK using the same regions as defined earlier. Finally, for differences in regional TSPO BP_ND_ or DKI metrics that remained statistically significant after FDR correction, we tested the correlation of individual regional imaging metrics with individual cognitive test results in our whole population using Pearson correlation after determining normal distribution. All statistical analyses were performed using R, version 4.1.1.

## Results

We performed neuropsychological evaluation on 32 patients, and included 27 matched patients with a >3-year history of aSAH. Patient characteristics are shown in Table 1. The median age of the included patients was 59 (range 34–73), and 18 patients (67%) were female. All patients were free of mild cognitive impairment or mood disorders prior to aSAH onset. No patients had prevalent inflammatory disease and none used immunomodulating medication. There were no statistically significant between-group differences in baseline characteristics. Results of the neuropsychological evaluations are shown in Supplemental Table 1. Fourteen patients had cognitive impairment and 13 no cognitive impairment.

**Table 1.** Characteristics of included patients.

	All patients (n = 27)	aSAH-CI (n = 14)	aSAH-NCI (n = 13)
Age (mean, range)	59 (34–73)	60 (34–72)	58 (41–73)
Females (n, %)	18 (67%)	8 (57%)	10 (77%)
Years after aSAH (mean, SD)	7.4 (2.5)	7.5 (2.2)	7.7 (1.8)
Aneurysm location (n, %)			
- ACA, ACOM	14 (52%)	7 (50%)	7 (54%)
- PCOM	3 (14%)	1 (7%)	2 (15%)
- ICA	1 (3%)	1 (7%)	0 (0%)
- MCA	8 (28%)	5 (36%)	3 (23%)
- Vertebral or basilar artery	1 (3%)	0 (0%)	1 (8%)
Focal infarction present (n, %)	15 (56%)	9 (64%)	6 (46%)
Rebleeding (n, %)	5 (19%)	2 (14%)	3 (23%)
DCI (n, %)	1 (4%)	1 (7%)	0 (0%)
Hydrocephalus (n, %)	6 (22%)	3 (21%)	3 (23%)
Prior mild cognitive impairment (n, %)^a^	12 (44%)	8 (57%)	4 (31%)
Days of hospital stay (median, range)	17 (12–62)	15 (12–62)	19 (14–40)
mRS at 3 months (median, range)	1 (0–4)	1 (0–4)	1 (0–3)

Diagnoses were made following aSAH but prior to study inclusion. Legends: aSAH = aneurysmal subarachnoid hemorrhage; CI = cognitive impairment; NCI = no cognitive impairment; SD = standard deviation; ACA = anterior cerebral artery; ACOM = anterior communicating artery; PCOM = posterior communicating artery; ICA = internal carotid artery; MCA = middle cerebral artery; DCI = delayed cerebral ischemia; mRS = modified Rankin Scale.

a No patients presented with mild cognitive impairment prior to aSAH.

### PET-imaging: validation of reference region

The mean BP_ND_ and corresponding 95% confidence interval (CI) of seven included patients and six historical controls, both at three different timepoints, are shown in Supplementary Figure 1. The average cerebellar gray matter SUV_BSA_ value across all timepoints was 0.52 [95% CI: 0.45; 0.59] in aSAH patients, and 0.53 [95% CI: 0.47; 0.59] in historical controls, suggesting cerebellar gray matter may be used as a reference region in the current study (Supplemental Figure 1).

### PET-imaging: differences in long-term neuroinflammation

**Figure 1.**
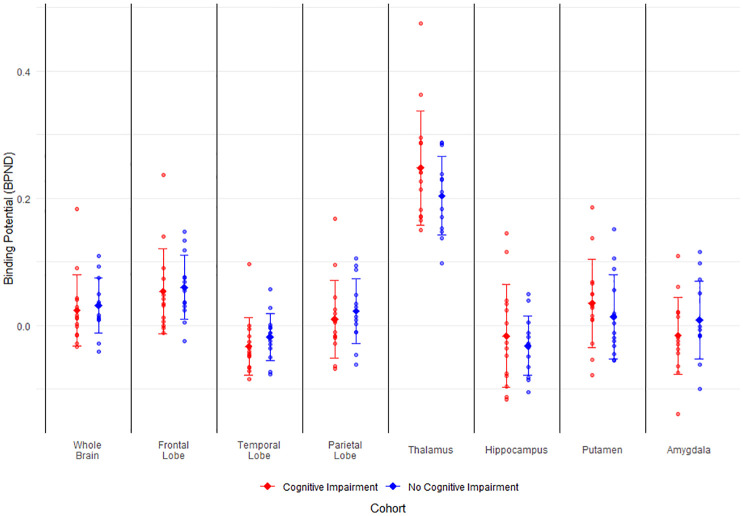
Binding potential of [^18^F]DPA-714 in regions of interest of patients > 3 years after aSAH. Binding potential of aSAH patients with and without cognitive impairment. Error bars represent mean and standard deviation. BP_ND_ = binding potential, aSAH = aneurysmal subarachnoid hemorrhage.

Whole-brain TSPO binding potential was similar in patients with and without cognitive impairment (mean BP_ND_ −0.046 [95% CI: −0.105; 0.013] vs −0.047 [95% CI: −0.108; 0.014], p = 0.98), and there were no differences in individual ROIs (Figure 1, Supplemental Table 2, Supplemental Figures 2 and 3). The sensitivity analysis excluding brain regions with infarction did not change our results (data not shown). The second sensitivity analysis in which we compared regional means of white matter TSPO BP_ND_ did not show statistically significant differences in white matter BP_ND_ in any of our included ROIs (Supplemental Figure 4). Regression of global cognitive scores onto regional BP_ND_ was statistically non-significant across all ROIs (data not shown).

### DKI: differences in kurtosis parameters in aSAH patients with and without cognitive impairment

**Figure 2.**
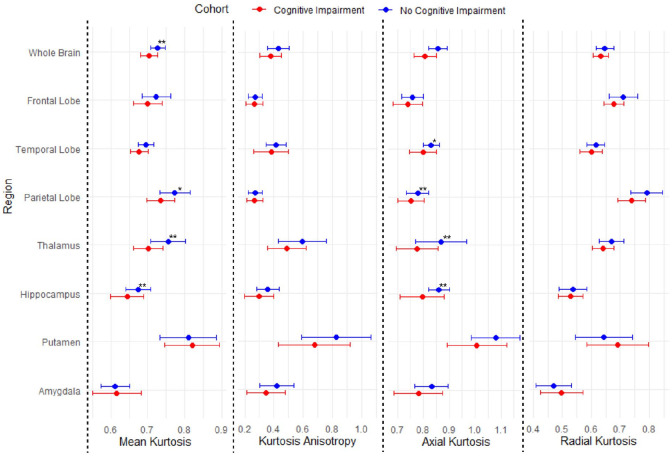
Kurtosis parameters in regions of interest of patients > 3 years after aSAH. Error bars representing mean and standard deviation of kurtosis parameters. aSAH = aneurysmal subarachnoid hemorrhage. *Represents p-value < 0.05. **Represents p-value < 0.05 after correction for false discovery rate.

Kurtosis parameters are shown in Figure 2 and Supplemental Table 3. Compared to participants without cognitive impairment, those with cognitive impairment had a lower whole-brain MK (mean MK 0.70 [95% CI: 0.69–0.72] vs mean MK 0.73 [95% CI: 0.72–0.74], corrected p = 0.03) and a lower whole-brain AK (mean AK 0.81 [95% CI: 0.78–0.83] vs mean AK 0.86 [95% CI: 0.84–0.87], corrected p = 0.04). Patients with cognitive impairment also had lower regional MK in the right thalamus (0.70 vs 0.74, corrected p = 0.02), left thalamus (0.71 vs 0.77, corrected p = 0.03), and left parietal lobe (0.73 vs 077, corrected p = 0.03), and regional AK was lower in the left hippocampus (0.80 vs 0.87, corrected p = 0.04) and left thalamus (0.77 vs 0.87, corrected p = 0.04). No statistically significant differences in KA or RK between those with and without cognitive impairment were observed after FDR correction.

### DKI: correlation between kurtosis parameters and individual cognitive tests

**Figure 3.**
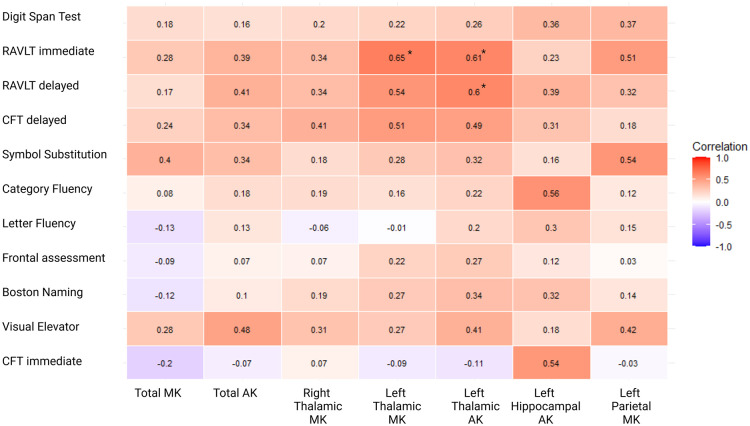
Correlations between cognitive tests and microstructural changes. Correlation matrix representing correlations between whole-brain or regional kurtosis parameters and individual cognitive tests. Numbers represent Pearson’s correlation coefficients. RAVLT = Rey Auditory Verbal Learning Task; CFT = complex figure test; MK = mean kurtosis; AK = axial kurtosis. *Represents p-value < 0.05 after correction for false discovery rate.

Figure 3 shows the correlations between whole-brain or regional kurtosis parameters and individual cognitive tests, for those parameters that differed between patients with and without cognitive impairment. Left thalamic MK correlated with RALVT immediate recall (r = 0.65, FDR corrected p < 0.01). Left thalamic AK correlated with RAVLT delayed immediate recall (r = 0.61, FDR corrected p = 0.01) and with RAVLT delayed recall (r = 0.60, FDR corrected p = 0.01). After FDR correction, no statistically significant correlations were found between any of the individual cognitive tests and total MK, total AK, right thalamic MK, or left parietal MK.

## Discussion

This study shows that the extent of microglial signal measured with [^18^F]DPA-714 PET-imaging did not differ between aSAH patients with and without long-term cognitive impairment. DKI revealed that microstructural brain damage is more pronounced in patients with than in those without cognitive impairment after aSAH and correlated with individual cognitive tests.

Studies using TSPO PET in other neurological diseases have shown varying results. In both acute and chronic ischemic stroke, TSPO binding was upregulated and related to microstructural white matter damage, but the extent and time course of upregulation differed between studies, potentially due to differences in pathophysiology or methodological differences (PET tracer used, analysis methods and timing of scanning in the disease course).^24,25^ In patients with traumatic brain injury (TBI), TSPO was upregulated more than 10 years after initial trauma and related to cognitive functioning.^26^ In patients with AD, studies have shown increased TSPO binding, which related to cognitive functioning,^27^ while others show no differences between AD patients and controls.^28^

After aSAH in mice, the number of microglia increases rapidly.^29^ In rodents, acute TSPO binding increases with SAH severity, and TSPO is related to upregulation of markers of activated microglia and astrocytes.^30^ Morphologically, these microglia assume a more pro-inflammatory state in the first days after aSAH, shifting to an anti-inflammatory state at a later stage.^29^ Our findings did not show evidence that long-term inflammation plays a role in the pathophysiology of cognitive impairment following aSAH. Many ROIs showed a BP_ND_ of around or below zero, suggesting there is no specific radiotracer uptake in these regions.

In our study, MK and AK were decreased in patients with cognitive impairment. DKI quantifies the non-Gaussian diffusion of water in tissue. MK and AK are thought to reflect tissue integrity at a microstructural level, including cellular density, neurite complexity, and the presence of glial cells.^31^ While these measures are sensitive to pathological changes, they are not specific to a single underlying cause and may be influenced by a range of processes, including neuroinflammation, neurodegeneration, or vascular pathology.^11^ Previous results have been reported for both patients with AD and patients with TBI, where MK is decreased and correlated with cognitive functioning, while in patients with TBI, this decrease is specific to the thalamus and internal capsule.^32,33^ In our study, the decreased kurtosis was most pronounced in the left thalamus, which correlated with performance on a test of verbal memory independently of neuroinflammation. This is in line with previous work, as the left thalamus has long been recognized as a key anatomical driver of performance on verbal memory tasks.^34^

In a study using longitudinal DTI, patients with cognitive impairment after aSAH showed a higher mean diffusivity (MD) than those without cognitive impairment after 2 weeks, contradicting our results.^35^ However, this study also reports a non-significant decrease of MD between 2 weeks and 6 months after aSAH. Given our findings that neuroinflammation was not increased, but kurtosis metrics were decreased and strongly correlation with cognitive performance, it is possible that an initial acute neuroinflammatory signal, which has since been resolved, has induced microstructural brain injury such as secondary thalamic degeneration, as previously observed in ischemic stroke.^36^ Future studies are necessary to determine whether this (sub)acute neuroinflammation plays a role in changes in white matter integrity, preferably using longitudinal data collection.

Our study has limitations. First, while many previous studies have suggested TSPO to be a marker of microglia activation, the increased expression of TSPO in activated microglia is unique to rodents and TSPO binding in humans represents the density of inflammatory cells rather than their activation state.^37^ Although we did not find a difference in the density of inflammatory cells, we cannot rule out that there might be a difference in their morphology or function. Second, while using a pseudo-reference region in TSPO PET analysis is common, the gold standard remains the use of plasma input compartmental models. However, due to the lack of blood data for the current study, we attempted to validate the use of cerebellum gray matter as reference region by comparing the SUV_BSA_ values with those from a previously collected cohort that used plasma input modeling.^20^ While this does not definitively confirm cerebellum as the ideal reference region, it suggests that cerebellum gray matter can serve as a pseudo-reference region. Third, we did not exclude patients with prevalent mood disorders that might affect cognitive functioning, nor did we exclude patients with prevalent inflammatory disease or using immunomodulating medication. While we confirmed that no patients had formal diagnoses of mood disorders or systemic inflammatory condition, and none used immunomodulating medication, it is possible that these diagnoses were made outside of the scope of our hospital records. Finally, we based the grouping of patients into cognitive impairment or not on a pragmatic neuropsychological battery, which might not be robust enough for a formal diagnosis of cognitive impairment. However, treating cognitive function as a continuous predictor did not alter our result, confirming that cognitive functioning was not associated with TSPO BP_ND._ Strengths of our study are the extent of our cognitive assessment, the inclusion of long-term aSAH survivors, and the use of multiple novel imaging modalities.

## Conclusion

Our results do not support the hypothesis that long-term cognitive impairment after aSAH is caused by long-term neuroinflammation. Instead, microstructural brain damage may play a role.

## Supplemental Material

sj-pdf-1-wso-10.1177_17474930251362004 – Supplemental material for Neuroinflammation in long-term cognitive impairment after aneurysmal subarachnoid hemorrhage Supplemental material, sj-pdf-1-wso-10.1177_17474930251362004 for Neuroinflammation in long-term cognitive impairment after aneurysmal subarachnoid hemorrhage by Reinier WP Tack, Nelleke Tolboom, Bas Meyer Viol, Sandeep SV Golla, Bart NM van Berckel, Irene C van der Schaaf, Ronald Boellaard, Alberto de Luca, Martine JE van Zandvoort, Johanna MA Visser-Meily, Elly M Hol, Gabriel JE Rinkel and Mervyn DI Vergouwen in International Journal of Stroke
