# Supplementary material for: Neuroinflammation in long-term cognitive impairment after aneurysmal subarachnoid hemorrhage
Source: Int J Stroke. 2025 Jul 15;20(10):1301–9. doi: 10.1177/17474930251362004 (PMC12664920; doi:10.1177/17474930251362004)
Supplement: sj-pdf-1-wso-10.1177_17474930251362004 – Supplemental material for Neuroinflammation in long-term cognitive impairment after aneurysmal subarachnoid hemorrhage [file sj-pdf-1-wso-10.1177_17474930251362004.pdf]

Supplementary Materials

Supplementary Table 1. 2

Results on neuropsychological evaluation 2

Supplementary Table 2. 3

Binding potential of [18F]DPA-714 in regions of interest 3

Supplementary Table 3. 4

Kurtosis metrics of aSAH survivors 4

Supplementary Figure 1. 5

Validation of reference region 5

Supplementary Figure 2. 6

TSPO PET in aSAH patients with cognitive impairment 6

Supplementary Figure 3. 7

TSPO PET in aSAH patients without cognitive impairment 7

Supplementary Figure 4. 8

Binding potential of [18F]DPA-714 in white matter of patients >3 years after aSAH 8

Supplementary Table 1.

Results on neuropsychological evaluation

Cognitive subtest	aSAH-CI<br>(n = 14)	aSAH-NCI<br>(n = 13)	P-value
Symbol substitution Test	7.8 +/- 3.2	10.6 +/- 2.6	0.02
WAIS 4: Digit Span Test	6.9 +/- 2.6	9.9 +/- 2.4	<0.01
Rey-Auditory Verbal Learning Test Dutch Immediate Recall (T-score)	43.5 +/- 11.4	58.2 +/- 7.0	<0.01
Rey-Auditory Verbal Learning Test Dutch Delayed Recall (T-score)	39.5 +/- 14.5	55.7 +/- 9.2	<0.01
Test of Everyday Attention (TEA): Visual Elevator Test (Accuracy score)	8.4 +/- 1.6	9.4 +/- 1.7	0.15
Boston Naming Test (score)	155.4 +/- 12.4	164.6 +/- 5.5	0.02
Verbal Fluency Test: phonemic (T-score)	48.4 +/- 7.4	52.9 +/- 12.1	0.26
Verbal Fluency Test: semantic (T-score)	59.4 +/- 15.9	78.0 +/- 19.5	0.01
Rey Complex Figure Test Immediate recall	36.7 +/- 1.5	36.4 +/- 1.1	0.55
Rey Complex Figure Test Delayed recall	21.3 +/- 6.1	26.1 +/- 5.8	0.04
Go-No Go of FAB	3 +/- 0.0	3 +/- 0.0	-

Cognitive Scores for both groups, numbers represent mean scores +/- standard deviation. Legends: WAIS 4: Wechsler Adult Intelligence Scale; FAB: Frontal Assessment Battery; aSAH = aneurysmal subarachnoid hemorrhage; CI: cognitive impairment; NCI: no cognitive impairment.

Supplementary Table 2.

Binding potential of [18F]DPA-714 in regions of interest

Region	Mean CI (n = 14)	SD	Mean NCI (n = 13)	SD	P-value	FDR
Total	-0.046	0.059	-0.047	0.061	0.974	0.974
Total GM only	0.024	0.056	0.031	0.043	0.705	0.899
Frontal Lobe Left GM	0.055	0.074	0.062	0.052	0.775	0.899
Frontal Lobe Right GM	0.053	0.061	0.058	0.052	0.822	0.899
Frontal Lobe Left WM	-0.148	0.058	-0.179	0.068	0.220	0.899
Frontal Lobe Right WM	-0.156	0.064	-0.171	0.078	0.612	0.899
Parietal Lobe Left GM	0.009	0.064	0.024	0.053	0.518	0.899
Parietal Lobe Right GM	0.011	0.062	0.021	0.054	0.640	0.899
Parietal Lobe Left WM	-0.187	0.051	-0.210	0.056	0.268	0.899
Parietal Lobe Right WM	-0.200	0.050	-0.210	0.057	0.633	0.899
Temporal Lobe Left GM	-0.035	0.047	-0.014	0.041	0.231	0.899
Temporal Lobe Right GM	-0.031	0.045	-0.022	0.037	0.596	0.899
Temporal Lobe Left WM	-0.193	0.040	-0.213	0.047	0.246	0.899
Temporal Lobe Right WM	-0.199	0.033	-0.206	0.055	0.693	0.899
Thalamus Left	0.253	0.086	0.196	0.062	0.059	0.899
Thalamus Right	0.246	0.111	0.212	0.066	0.353	0.899
Putamen Left	0.035	0.057	0.007	0.064	0.244	0.899
Putamen Right	0.038	0.093	0.024	0.075	0.675	0.899
Hippocampus Left	-0.015	0.087	-0.017	0.046	0.944	0.971
Hippocampus Right	-0.021	0.090	-0.049	0.060	0.350	0.899
Amygdala Left	-0.018	0.068	0.021	0.054	0.117	0.899
Amygdala Right	-0.013	0.069	-0.023	0.135	0.811	0.899

Regional binding potential stratified by laterality. Legend: CI: Cognitive Impaired; NCI: Non Cognitive Impaired; GM: Grey Matter; WM: White matter; FDR: false discovery rate

Supplementary Table 3.

Kurtosis metrics of aSAH survivors

Region of Interest	MK CI	MK NCI	KA CI	KA NCI	AK CI	AK NCI	RK CI	RK NCI
Total	0.70*	0.73*	0.38	0.43	0.81*	0.86*	0.63	0.65
Frontal Lobe Left	0.70	0.73	0.26	0.27	0.74	0.76	0.69	0.72
Frontal Lobe Right	0.70	0.72	0.27	0.27	0.74	0.76	0.67	0.70
Parietal Lobe Left	0.73*	0.77*	0.27	0.28	0.75	0.78	0.73	0.79
Parietal Lobe Right	0.74	0.77	0.26	0.26	0.75	0.77	0.75	0.79
Temporal Lobe Left	0.67	0.69	0.38	0.41	0.79	0.83	0.60	0.62
Temporal Lobe Right	0.68	0.70	0.39	0.41	0.80	0.83	0.60	0.61
Thalamus Left	0.71*	0.77*	0.46	0.57	0.77*	0.87*	0.64	0.68
Thalamus Right	0.70*	0.74*	0.52	0.62	0.78	0.87	0.64	0.66
Putamen Left	0.83	0.82	0.65	0.83	1.01	1.10	0.70	0.64
Putamen Right	0.81	0.80	0.70	0.82	1.01	1.06	0.69	0.64
Hippocampus Left	0.65	0.67	0.28	0.34	0.79*	0.85*	0.54	0.54
Hippocampus Right	0.64	0.68	0.32	0.38	0.80	0.87	0.52	0.54
Amygdala Left	0.63	0.61	0.31	0.40	0.78	0.83	0.52	0.47
Amygdala Right	0.60	0.61	0.39	0.44	0.78	0.83	0.48	0.47

Regional diffusion kurtosis metrics stratified by laterality. Numbers represent mean values. *Represents $p < 0.05$ after false discovery correction. Legend: aSAH: Aneurysmal Subarachnoid Hemorrhage; MK: Mean Kurtosis; KA: Kurtosis Anisotropy; AK: Axial kurtosis; RK: Radial Kurtosis; CI: Cognitive Impairment; NCI: No Cognitive Impairment.

Supplementary Figure 1.

Validation of reference region

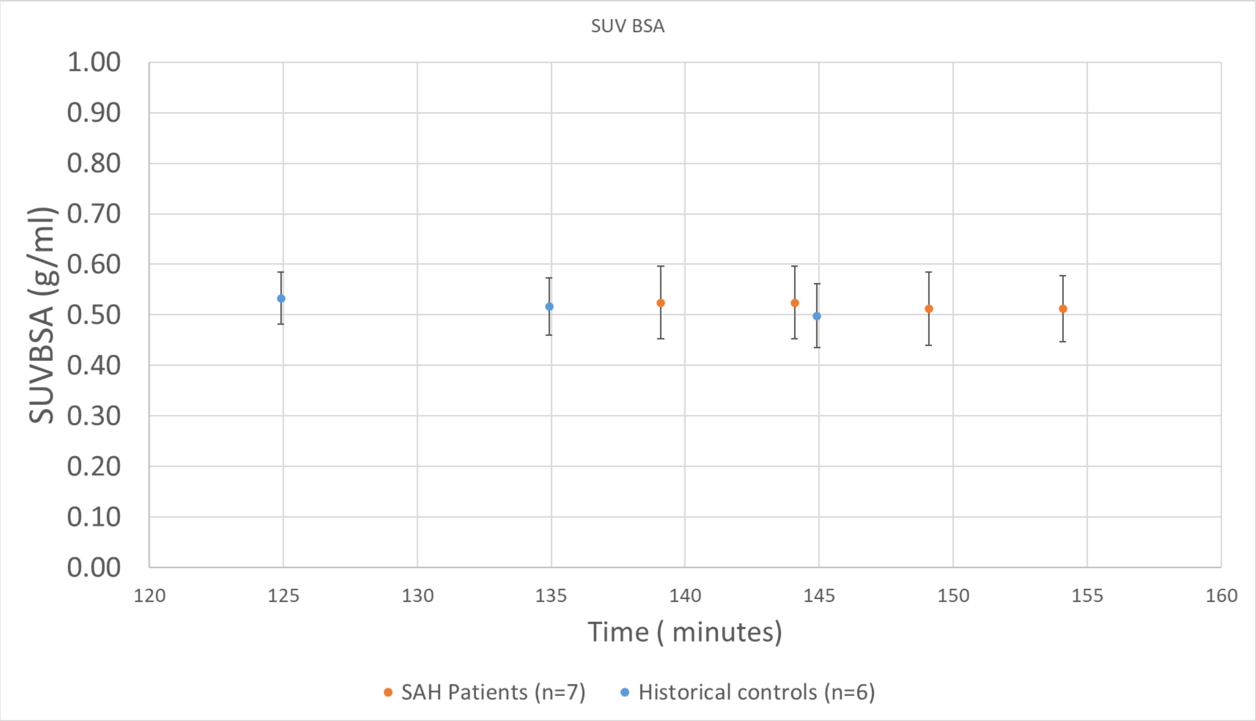

Mean standardized uptake values across different timepoints for aSAH patients and historical cohorts. Errors represent mean and standard deviation for entire group (patients or controls) at different time points. Legend: SUV: Standardized uptake value; BSA: body surface area.

Supplementary Figure 2.

TSPO PET in aSAH patients with cognitive impairment

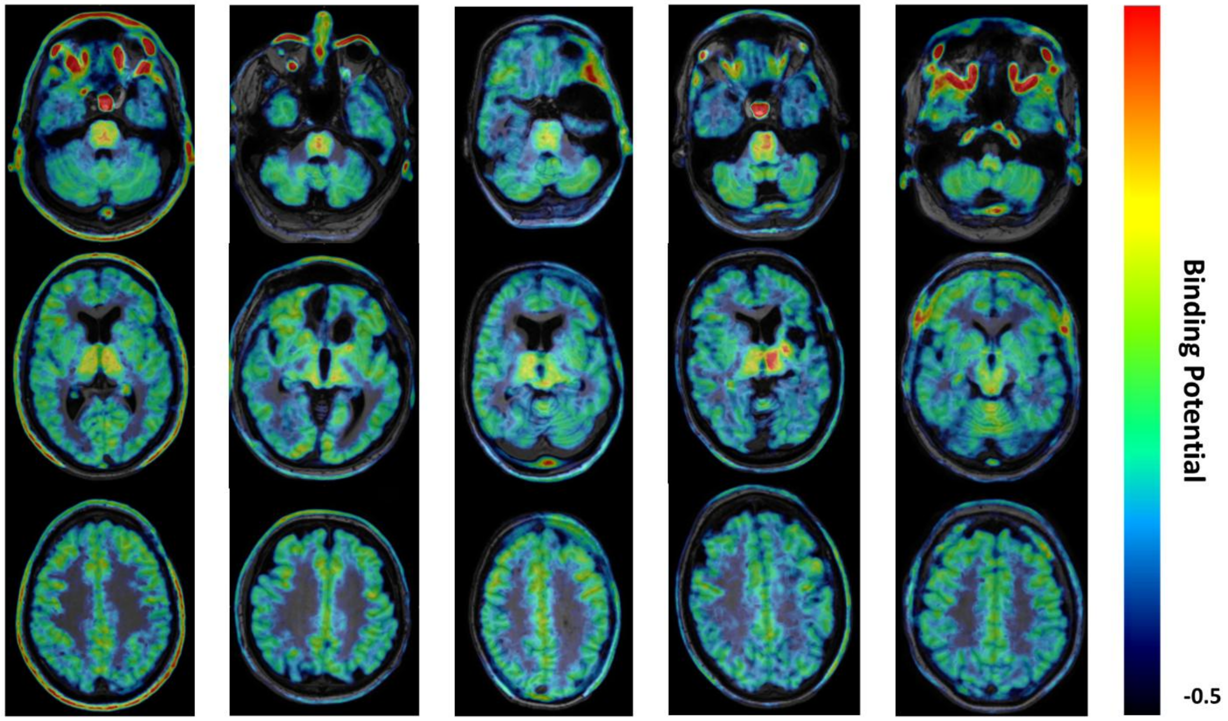

Examples of TSPO PET images for 5 patients with cognitive impairment after aSAH. For each patient, 3 coupes are shown.

Supplementary Figure 3.

TSPO PET in aSAH patients without cognitive impairment

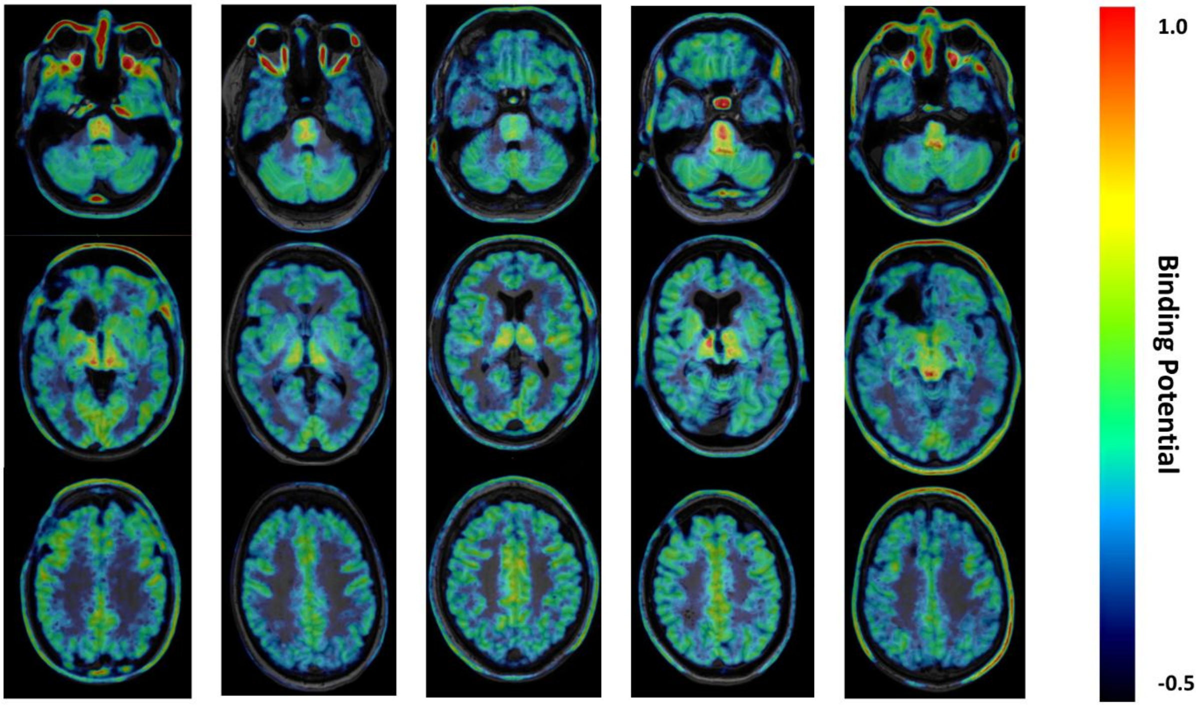

Examples of TSPO PET images for 5 patients without cognitive impairment after aSAH. For each patient, 3 coupes are shown.

Supplementary Figure 4.

Binding potential of [18F]DPA-714 in white matter of patients >3 years after aSAH

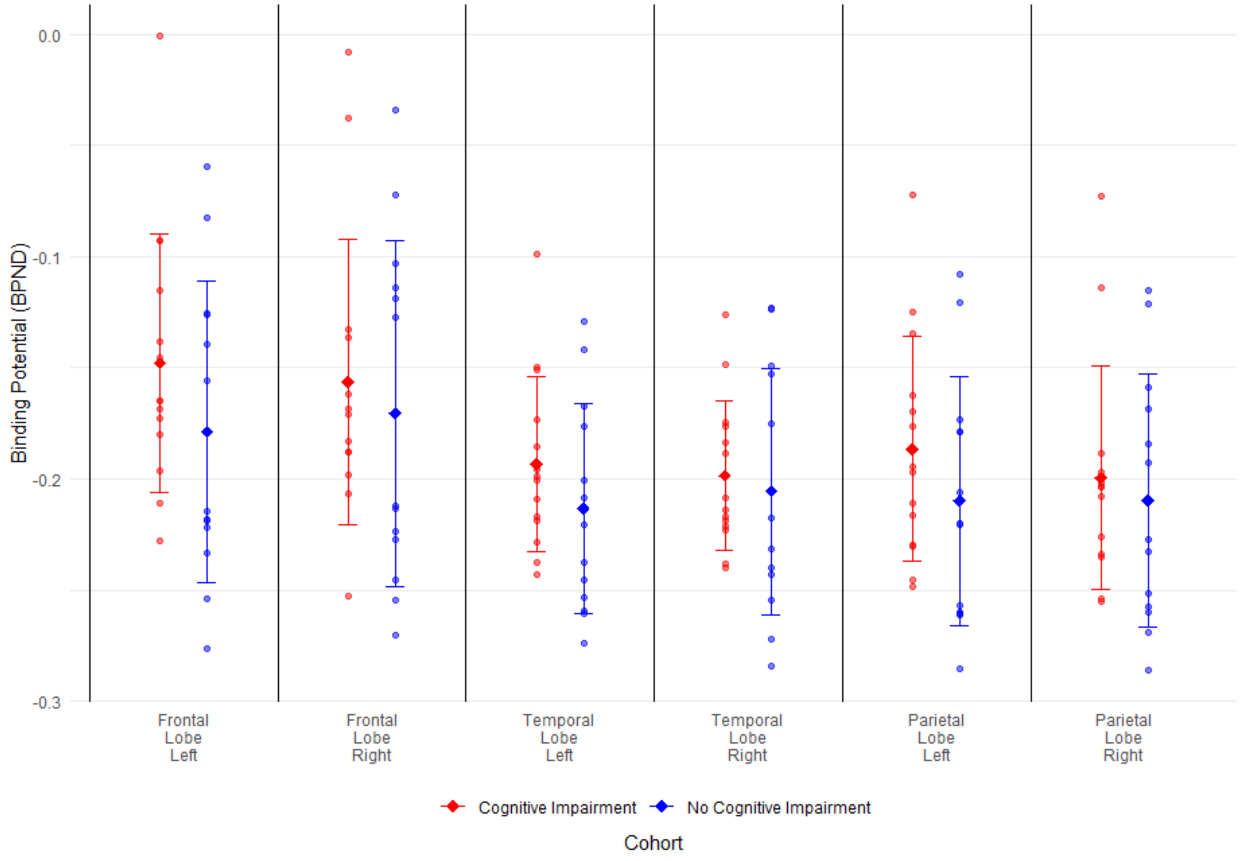

White matter binding potential of aSAH patients with and without cognitive impairment, stratified by laterality. Error bars represent mean and standard deviation. Legends: BPND = binding potential, aSAH = aneurysmal subarachnoid hemorrhage.
